# The Case against a Smoker's License

**DOI:** 10.1371/journal.pmed.1001343

**Published:** 2012-11-13

**Authors:** Jeff Collin

**Affiliations:** Global Public Health Unit, School of Social & Political Science, University of Edinburgh, Edinburgh, Scotland, United Kingdom

## Abstract

Tobacco continues to kill millions of people around the world each year and its use is increasing in some countries, which makes the need for new, creative, and radical efforts to achieve the tobacco control endgame vitally important. One such effort is discussed in this *PLOS Medicine* Debate, where Simon Chapman presents his proposal for a “smoker's license” and Jeff Collin argues against. Chapman sets out a case for introducing a smart card license for smokers designed to limit access to tobacco products and encourage cessation. Key elements of the smoker's license include smokers setting daily limits, financial incentives for permanent license surrender, and a test of health risk knowledge for commencing smokers. Collin argues against the proposal, saying that it would shift focus away from the real vector of the epidemic—the tobacco industry—and that by focusing on individuals it would censure victims, increase stigmatization of smokers, and marginalize the poor.

## Abstract 2

In one half of a *PLOS Medicine* Debate, Jeff Collin argues against Simon Chapman's proposal for a smoker's license, saying that it is strategically flawed and ethically unsound.

Despite its many successes, the need for tobacco control advocates to think outside the box and explore radical new options remains compelling. As always it is difficult to know whether to be more impressed by the scale of recent legislative achievements in driving change, or to despair at the widespread persistence of damaging behaviours and devastating health impacts. For all the progress made, with increasing numbers of countries implementing the diverse evidence-based measures recommended by the WHO Framework Convention on Tobacco Control (FCTC), no state has managed to reduce smoking prevalence to an extent that anyone in public health might regard as tolerable. The attainment of a tobacco-free future, so critical to any global conception of health for all, remains elusive. The need to rethink and extend tobacco control's playbook is therefore clear, and the recent upsurge of interest in endgame strategies [1]–[3] is both welcome and necessary.

In this context, Simon Chapman's typically powerful advocacy of the case for a smoker's license in this issue of *PLOS Medicine* offers an important contribution to emerging debates [4]. While it seems reasonable to hypothesize that such a scheme could further reduce tobacco consumption in some countries, the notion of licensing smokers raises significant strategic concerns and highlights broader questions of principle with which tobacco control must engage. In searching for new measures to drive towards an endgame, Chapman's proposal highlights the importance of reflecting not only on what new legislative wins might be within reach for tobacco control advocates, but to more carefully delineate the ethical limits of pursuing such goals.

In critiquing the smoker's license proposal, I should acknowledge that in some specific national contexts its adoption may be comparatively unproblematic (Chapman notes its similarities with ideas under discussion in Singapore), while there are clearly context-specific aspects to the objections outlined below. In countries where digital ID cards are routinely carried or objections to authorities holding data are limited, for example, linking tobacco purchases to such cards may be largely unproblematic technically or politically; at least some of the data envisaged under this scheme may be generated; and the idea of progressively raising the legal age of purchase merits wider consideration.

In highlighting the problems associated with the ubiquity of tobacco retail outlets, Chapman importantly recognises the continuing comparative neglect of supply-side issues. It is indeed an historical absurdity that so dangerous a product should be so readily available, and the policy implications of “tobacco sale (being) subject to trivial controls compared with other dangerous products” merit exploration. While point-of-sale display bans [5],[6] are beginning to focus attention on regulating the retail environment, it is disappointing that more sustained attention hasn't yet been given to models of controlling availability. In this respect, at least, Chapman's analogy with restricted access to medicines has some merit, while studies of the hours of sale and density of outlets for alcohol suggest that measures to reduce the availability of harmful products can contribute effectively to public health strategies [7],[8]. But this literature also suggests that the ubiquity of tobacco products can be challenged by rather different routes than that specified in the smoker's license scheme, and Chapman's proposal is curious in seeking to reshape the structure of retailing environment via measures that are so starkly targeted towards consumers.

It is against this radical shift of regulatory attention to a direct focus on smokers that fundamental objections to the scheme should be directed. To date the tobacco control agenda has been principally concerned with regulating the conduct of the tobacco industry, on the basis of an understanding that effectively curbing this industrial epidemic [9] is best achieved via actions that tackle the disease vector [10]. The unique centrality of this perspective within tobacco control, embedded in the WHO FCTC, has been crucial to recent successes and contrasts starkly with the continuing scope for partnership with industry that widely typifies alcohol and obesity policies at national and international levels [11]. It also indicates a policy agenda that is far from exhausted. From a global perspective, the challenges of effectively implementing the FCTC centre on industry opposition, exacerbated by tensions between public health goals and trade liberalisation via the World Trade Organisation and, increasingly, bilateral agreements [12]. Even among those comparatively successful countries to which Chapman directs his proposal, key priorities include the development of approaches to taxation that can more effectively target industry profitability, ensuring that health objectives aren't undermined via product innovation in smokeless tobacco and nicotine delivery devices such as e-cigarettes, and building on progress in Australia to ensure the adoption and implementation of generic packaging. There is also an important need for more creative thinking in how we regulate the industry, which should centre on changing a system of manufacture and promotion of such harmful products centred on the corporation, an institution that is staggeringly ill-suited to such roles when viewed from a public health perspective [13],[14].

It is particularly important that the search for innovative new strategies doesn't rather create gifts for the tobacco industry, which this one undoubtedly would. It is arguably no more radical a proposal for social change than that represented by Ireland's legislation for smoke-free work places less than a decade ago. Yet while the implications of such change for smokers have been profound, such reforms have been recognised as legitimate and attracted broad consent since they effectively reconciled innovative health protection with broader norms. Smoke-free policies have been recognised and understood as unambiguously liberal measures rather than authoritarian intrusions on personal freedom. In advancing a case focused on the protection of non-smokers, workers, and children, such legislation conforms to JS Mill's classic formulation of the harm principle in *On Liberty*: “(t)he only purpose for which power can be rightfully exercised over any member of a civilized community, against his will, is to prevent harm to others” [15]. The coherence of such positions has been of great strategic importance, not least in convincingly rebutting the oft-repeated charges of “health fascism” made by tobacco companies and their front groups [16].

The authoritarian connotations of the smoker's license would inevitably meet with broad opposition. In the United Kingdom, for example, successive governments have failed to introduce identity cards. If it's very difficult to envisage health advocates securing support for a comparable scheme on the basis of a public health rationale, it is still harder to see why they should wish to.

In constituting “an explicitly user-focused form of regulation” [4], Chapman's proposal to license smokers has the potential to dramatically exacerbate their stigmatization. That many smokers would feel that they were “being treated like registered addicts” [4] seems inevitable, and indeed is central to the scheme's design. Chapman is correct to note that many industry- and product-focused measures do directly affect smokers, but he acknowledges that what is being proposed here is qualitatively different. Given the pronounced social gradient of smoking, core tobacco control measures have long had implications for the poor (most obviously via the use of taxation to reduce consumption); a distinguishing feature of this scheme is that, in effect, it would be censuring the poor.

In developing new strategies to further reduce tobacco consumption, we need to seek to better manage the central tension between smoking and stigma. While public health generally sees stigmatization as inimical to its goals, tobacco control has demonstrated the capacity of “efforts to denormalize, marginalize and stigmatize smoking” to “further the goals of public health” [17]. But this does not mean that tobacco control should always view increased stigmatization as a price worth paying for reduced consumption. The proposal to require licenses will inevitably be widely perceived as demeaning, onerous, and punitive, and in explicitly targeting smokers would dramatically exacerbate the sense that smoking “just has that sort of feel about it, a leper” [18].

A fundamental challenge confronting any endgame strategy is that the move towards a tobacco-free society should address the social determinants of health and promote equity and social justice. The proposal for a smoker's license should be rejected as failing this challenge.
